# Relative Role of ON/OFF Pathways in Resolving and Adapting to Blur in Myopes and Emmetropes

**DOI:** 10.1167/iovs.66.9.29

**Published:** 2025-07-11

**Authors:** Hosein Hoseini-Yazdi, Andrew Carkeet, Rida Ahmed, Faaizah Ali, Julia Anderson, Zhi Yu Chan, Jade Chng, Nitansha Nand, Anh Pham, Hannah Soen, Leeanne Su, Mandy Truong, Scott A. Read

**Affiliations:** 1 Contact Lens and Visual Optics Laboratory, Centre for Vision and Eye Research, Optometry and Vision Science, Queensland University of Technology, Brisbane, Queensland, Australia; 2 Centre for Vision and Eye Research, Optometry and Vision Science, Queensland University of Technology, Brisbane, Queensland, Australia

**Keywords:** adaptation, myopia, ON/OFF pathways, refractive error, visual acuity

## Abstract

**Purpose:**

To investigate the contribution of ON/OFF pathways to resolution in the presence of blur and adaptation of the human eye to blur.

**Methods:**

Twenty-three healthy young adults (24 ± 4 years) participated, including 11 myopes and 12 emmetropes exhibiting mean spherical equivalent refractive errors of −1.5 ± 0.7 diopters (D) and −0.02 ± 0.2 D, respectively. Visual acuity (VA) was examined over 30 minutes of exposure to +1.00 D myopic blur while overstimulating the ON pathway by viewing a chart displaying brighter letters than background, or the OFF pathway by viewing a luminance- and contrast-matched chart displaying darker letters than background. Linear mixed models examined the blur-induced VA loss and the subsequent relative changes in logMAR VA over time (i.e., blur adaptation), associated with ON/OFF pathways and refractive group.

**Results:**

The blur-induced VA loss was significantly less when overstimulating the ON pathway (0.33 ± 0.03 logMAR) compared to the OFF pathway (0.43 ± 0.03 logMAR; *P* < 0.001), with no significant difference between refractive groups (*P* = 0.79). Significant blur adaptation was also observed (*P* < 0.001), which was greater during ON-pathway overstimulation (−14% ± 2%) compared to OFF-pathway overstimulation (−9% ± 2%). Blur adaptation was significantly greater in emmetropes (−18% ± 3%) than myopes (−4% ± 3%; *P* = 0.004), particularly during ON-pathway overstimulation (interaction effect *P* = 0.008).

**Conclusions:**

Short-term overstimulation of the ON pathway was associated with a smaller blur-induced worsening in VA than overstimulation of the OFF pathway but yielded a greater blur adaptation response, which was more pronounced in emmetropes. Future studies are necessary to examine whether deficient blur adaptation in the ON pathway could serve as a psychophysical biomarker of myopic eye growth.

Visual acuity (VA) informs on the integrity of central vision responsible for resolving fine details in daily tasks and varies with optical imperfections related to defocus and higher order aberrations, pupil size, illumination, and luminance contrast.^1^^,^^2^ Neuronal factors also play a crucial role, with the limit of visual resolution determined by the anatomical separation of ON and OFF midget retinal ganglion cells, which mediate light increments and decrements, respectively.^3^^–^^5^ Anatomical and electrophysiological studies suggest that the receptive fields are smaller in the OFF compared to ON retinal ganglion cells, thus resulting in finer OFF-mediated spatial resolution.^6^ However, inconsistent and sometimes conflicting results have been reported regarding the impact of ON/OFF pathways on psychophysical measures of VA, with some studies reporting better VA with standard polarity charts (OFF-pathway overstimulation),^7^^–^^16^ whereas others have reported better VA with reversed polarity charts (ON-pathway overstimulation)^7^^–^^11^ or no difference in VA between chart polarities.^12^^–^^14^

Blur adaptation is the improvement in VA that follows a sustained period of optical blur that is not explained by any accompanying biometric or refractive changes in the eye.^17^^–^^25^ Earlier studies suggested that the reduced perception of blur following blur adaptation was primarily associated with reduced contrast sensitivity at lower spatial frequencies over time, passively leading to a more balanced contrast sensitivity across lower and higher spatial frequencies.^18^^,^^26^ However, more recent research has revealed active mechanisms driving blur adaptation, characterized by differential gain adjustments in the signaling of spatial frequency channels, with decreased contrast sensitivity at low spatial frequencies (0.5–1 cycles per degree [cpd]) and increased contrast sensitivity at medium (3–4 cpd) and high (8–12 cpd) spatial frequencies over time.^29^^,^^61^ These spatial frequency–dependent gain control adjustments in contrast sensitivity occurring at both retinal^27^^–^^30^ and cortical levels^31^^,^^32^ are thought to serve as the primary driver of VA improvements in blur adaptation. It has been suggested that neural pathways involving both dopaminergic ON bipolar and horizontal cells contribute to the retinal mechanisms of blur adaptation.^33^^–^^35^ Given that myopes typically demonstrate a better uncorrected VA compared to emmetropes who are exposed to the same level of image blur^24^^,^^36^ and some evidence suggesting a greater level of blur adaption in myopes than emmetropes,^20^^,^^22^ it is possible that blur adaptation is linked to the mechanisms involved in myopia development.

The relative activation of retinal ON cells and OFF cells, which respond to objects brighter or darker than the background, respectively, may play a key role in regulating eye growth and myopia development.^37^^–^^45^ Animal studies suggest that overstimulation of the OFF pathway or deficiencies in the ON pathway contribute to myopic eye growth.^38^^–^^41^ Recent research in humans also indicates that visual stimuli that overstimulate the OFF and ON pathways lead to consistent short-term changes in choroidal thickness that are considered biomarkers of longer term processes leading to myopia and emmetropia, respectively.^41^^–^^43^ Electrophysiological assessments of ON/OFF pathways in humans have also revealed differences between emmetropes and myopes, consistent with a deficit in ON pathway signaling in myopes,^44^^,^^45^ particularly at higher illuminations.^44^ On the other hand, psychophysical studies have revealed reduced contrast sensitivity in myopes compared to emmetropes, particularly under conditions that overstimulate the OFF pathway at mid-spatial frequencies.^46^ However, the relative influence of ON/OFF pathways on blur adaptation has not been previously explored, as studies of blur adaptation in humans have used standard high-contrast VA charts with black letters on a white background, primarily overstimulating the OFF pathway.^18^^–^^21^^,^^23^^,^^24^

This study aimed to investigate the role of ON/OFF pathways in resolving and adapting to blur and how these changes are affected by myopia. It is speculated that mechanisms for resolution with blur and blur adaptation may involve retinal dopamine,^33^^,^^34^ which plays a key role in high-resolution vision^35^^,^^47^^–^^49^ and is primarily expressed from the cone-mediated ON bipolar pathway.^50^ We therefore hypothesized that overstimulating the ON pathway using a VA chart with letters brighter than the background would result in less blur-induced decline in VA and greater blur adaptation compared to OFF-pathway overstimulation using a standard VA chart with letters darker than the background. Additionally, given the suggested link between the ON/OFF signaling and myopia development, it was hypothesized that ON-pathway overstimulation would lead to less VA decline with blur and greater blur adaptation in emmetropes than in myopes. Pupil size was also measured in the current study, as it is regulated by ON pathway signaling and has been shown to be affected in myopia.^44^ Given that blur adaptation is primarily driven by neural mechanisms,^17^^,^^19^^,^^22^ we hypothesized that optical factors associated with changes in pupil size would not significantly contribute to the improvements in VA following blur adaptation.

## Methods

### Participants

Twenty-three healthy young adults with normal best-corrected vision of logMAR 0.00 or better in each eye and normal binocular vision were recruited. No participant had any history or evidence of amblyopia, strabismus, accommodation dysfunction, or any ocular disease, injury, or surgery. Smokers and individuals using systemic or topical medications were excluded. All participants provided written informed consent to participate and were treated in accordance with the tenets of the Declaration of Helsinki. The study was approved by the Queensland University of Technology human research ethics committee.

Refractive status was determined by non-cycloplegic subjective refraction with maximum plus for best VA followed by binocular balancing. To reduce the potential for differences in blurred retinal image size (associated with the correcting and blur-inducing trial lenses) affecting the blur adaptation in myopes compared to emmetropes, only low myopic participants (spherical equivalent refraction [SER] of −2.50 to −0.50 D) showing less than 1.25 D anisometropia were included. None of the myopic participants was using rigid contact lenses or myopia control treatments. Myopic participants wearing soft contact lenses (*n* = 6) abstained from contact lens wear on the testing days, wore their optimal spectacle correction beforehand, and were corrected with trial lenses during the experimental visits. Therefore, complications associated with contact lens wear, such as variations in tear film quality during lens wear, were unlikely to affect the visual performance of myopes compared to emmetropes in this study. Because blur adaptation may be affected by habitual exposure to blur, only myopes who were optically corrected on a full-time basis were included, and individuals with more than 0.5 D of habitual under- or over-correction were excluded as determined by non-cycloplegic subjective refraction.

### Procedures

**Figure 1.**
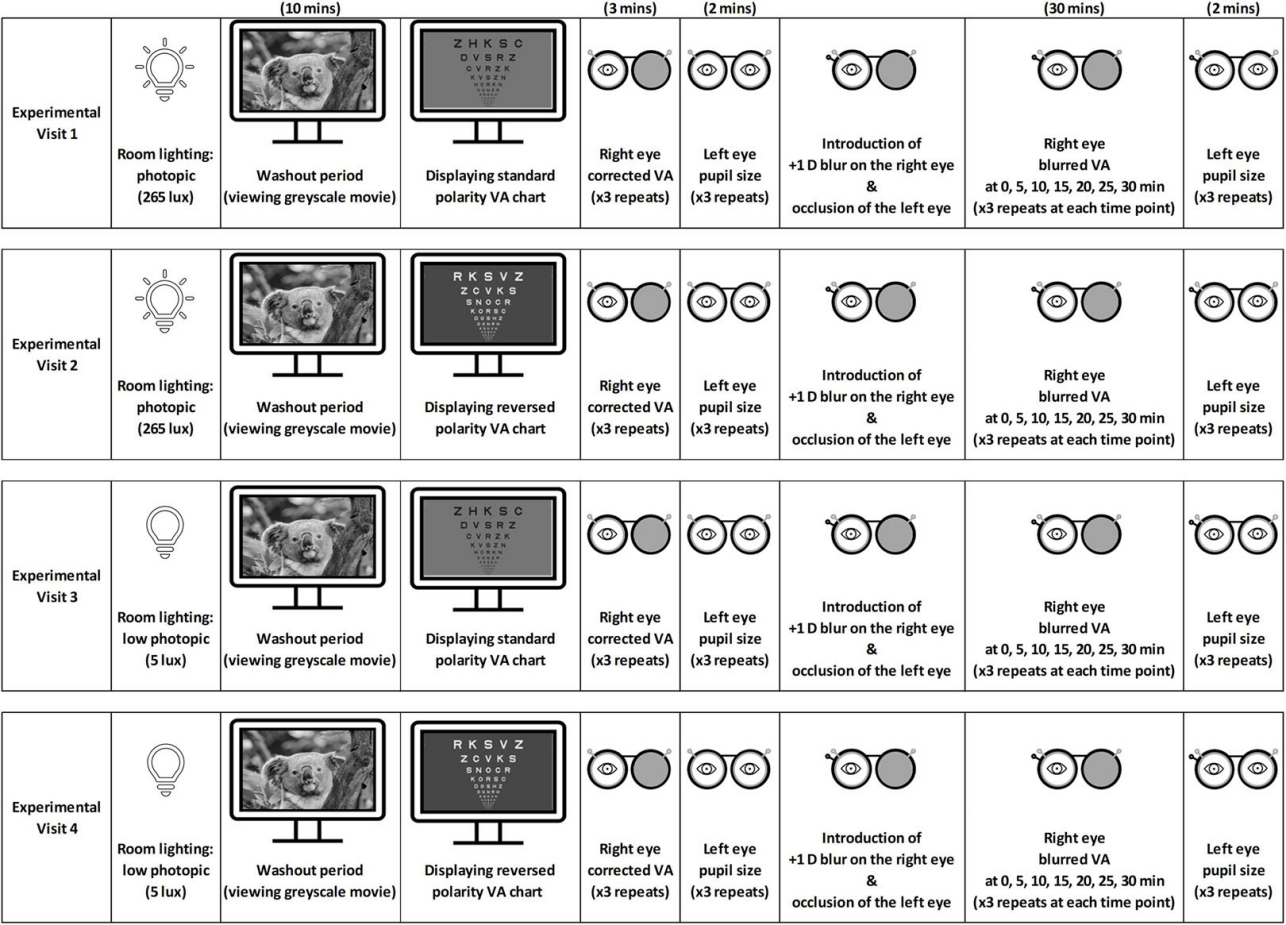
Overview of the four experimental visits to examine the blur adaptation response of the right eye to +1 D of myopic defocus over 30 minutes. Blur adaptation was examined during overstimulation of the OFF pathway by using a standard-polarity VA chart displaying darker letters than the background, as well as during overstimulation of the ON pathway by using a reversed-polarity VA chart displaying brighter letters than the background, under photopic (265 lux) and low photopic (5 lux) ambient illumination. Note that the experimental visits were conducted on separate days in a randomized order. The OFF- and ON-stimulating VA charts were matched in terms of the mean screen luminance and the Michelson luminance contrast of the letters, to ensure that differences in the task overall luminance and contrast did not confound the VA outcomes.

Eligible participants attended four randomly ordered study visits, scheduled between 9 AM and 4 PM at approximately the same time of day for each participant, during which +1 D blur was imposed monocularly on the optimally corrected right eye. VA was measured before, immediately after, and then every 5 minutes during blur exposure over 30 minutes (Fig. 1). The visits were at least 2 days apart to minimize any potential carryover effects.^51^

Each visit began with a 10-minute washout period of watching a grayscale movie at a 4-meter distance, presumably with an unbiased stimulation of the ON/OFF pathways; both eyes were optimally corrected using spherocylindrical trial lenses followed by measurement of corrected monocular VA. The +1 D blur was then induced on the right eye for 30 minutes using trial lenses with the left eye occluded. Because only participants with normal binocular vision and accommodation were included, it was unlikely that the transition from binocular viewing during the washout period to monocular viewing during the blur period had any significant impact on measures of monocular VA. During the blur exposure, participants were instructed to fixate on an optotype in a line that was two lines larger than the threshold blurred VA, presented either on an OFF-overstimulating (letters darker than background) or an ON-overstimulating (letters brighter than background) high-contrast logMAR VA chart. The charts were matched for overall luminance to minimize variations in pupil size between conditions. The VA chart was presented at 4-meter distance on a high-resolution computer monitor (28-inch UE590 UHD 4K, 3840 × 2160 resolution, 60-Hz refresh rate, subtending visual angles of 9° horizontally and 5° vertically; Samsung, Suwon, South Korea), generated by custom written software. Because the responses to ON/OFF overstimulation may vary between emmetropes and myopes with levels of ambient illumination,^44^ the OFF- and ON-overstimulating conditions were each assessed under photopic (265 lux) and low photopic (∼5 lux) illumination as measured with an FX-200 illuminometer (Watt Stopper, Santa Clara, CA, USA) at the eye plane facing toward the VA display monitor (Fig. 1).

Blur adaptation was examined using logMAR VA, assessed before exposure and then every 5 minutes during blur exposure, using the same OFF- and ON-stimulating charts that were used for the blur adaptation. Each VA measurement lasted approximately 1 minute, with the three repetitions taking around 3 minutes in total. The Michelson luminance contrast of the letters was 86%, and the mean luminance of the charts was 68 cd/m^2^, measured with a BM-7 luminance colorimeter over a 2° visual angle (Topcon, Tokyo, Japan). The letter and background luminance values were 6 cd/m^2^ and 77 cd/m^2^, respectively, for the OFF-stimulating chart, and they were 347 cd/m^2^ and 26 cd/m^2^, respectively, for the ON-stimulating chart. The luminance of the grayscale movie presented during the washout period varied randomly throughout the presentation and across different visits (ranging between 2 and 60 cd/m^2^). However, the same display screen was used for presenting both the grayscale movie and the VA charts, with various display characteristics, including overall screen brightness and contrast, maintained at similar levels.

The VA chart followed the letter and line spacing criteria used in Early Treatment Diabetic Retinopathy Study (ETDRS) and Bailey–Lovie charts,^52^ featuring multiple rows in a logarithmic progression format, with each row consisting of five Sloan letters. The letters were randomly changed to avoid any learning effects influencing subsequent VA measurements. Participants were instructed to identify each letter and read from the top row down the VA chart with responses entered by the examiner using a keyboard. The presentation of multiple rows of letter optotypes, rather than a single row near the threshold as used in adaptive staircase methods, ensured that the mean luminance of the screen and the range of letter sizes remained consistent across different study conditions and refractive groups. The program automatically terminated the measurement when three mistakes were made on a line.^53^ VA was measured using the letter-by-letter scoring^52^ and repeated three times at each time point.

Pupil size was measured in the left eye as an indirect estimate of the pupil size of the right eye to ensure that the exposure of the right eye to both the blur and the ON/OFF stimuli remained uninterrupted. This measurement was repeated three times and averaged both before and after the 30-minute blur adaptation of the right eye, using a VIP-300 Pupillometer (NeurOptics, Irvine, CA, USA) (Fig. 1). As only healthy subjects with normal pupil responses were included in the study, the pupil size of the left eye was primarily governed by consensual pupil responses and therefore reflected the pupil size of the right eye. It is unlikely that the prior occlusion of the left eye had any meaningful effect on this measurement.

### Statistical Analysis

The three repeated VA measurements collected at each time point were first screened to ensure that the within-subject standard deviation (*S_w_*) was less than 0.1 logMAR, thus within the reported coefficient of repeatability for measures of high-contrast VA in healthy adults.^54^^,^^55^ Any measurements causing *S_w_* to exceed this threshold were excluded. Three outcome measurements were derived from the monocular VA data: corrected logMAR VA prior to blur exposure, blur-induced VA loss (BIVAL, calculated as the immediate loss in logMAR VA due to blur relative to the corrected VA), and relative blur adaptation, calculated as the percentage change in logMAR VA during blur exposure relative to the initial BIVAL, as follows:

$$\begin{array}{ccc} & & \;\mathrm{Relative}\;\mathrm{blur}\;\mathrm{adaptation}\;\\ & & \;=\frac{\begin{array}{c} (Blurred\;VA\;at\;the\;\mathrm{beginning}\;\mathrm{of}\;\mathrm{blur}\\ period\;-\;\mathrm{Blurred}\;VA\;\mathrm{at}\;\mathrm{the}\;\mathrm{end}\;\mathrm{of}\;\mathrm{blur}\;\mathrm{period}) \end{array}}{BIVAL} \end{array}$$

All statistical analyses were carried out using SPSS Statistics 29 (IBM, Chicago, IL, USA). To examine the effect of illumination and contrast polarity on corrected VA, BIVAL, baseline pupil size, and the percentage change in pupil size following blur period relative to baseline pupil size, separate linear mixed models (LMMs) were applied. Each model included illumination, contrast polarity, refractive group, and all possible factorial interactions as fixed factors. A separate LMM was used to analyze relative blur adaptation, involving illumination, contrast polarity, time, refractive group, and all possible interactions with time as fixed factors. In all LMMs, a compound symmetry covariance structure was assumed for the repeated factors. Individual slopes and intercepts were also included as random factors, assuming a variance component covariance structure. Bonferroni-corrected post hoc tests were conducted for the significant main effects and interactions.

Repeated-measures analysis of covariance (ANCOVA) was conducted to examine the association between individual percentage changes in pupil size and BIVAL, as well as changes in relative blur adaptation. The results are presented as mean ± standard error of the mean (SEM). Positive changes in logMAR and relative blur adaptation indicate a decline in VA, whereas negative changes signify an improvement in VA.

## Results

The mean ± SD age of the participants was 24 ± 4 years (range, 19–32 years). Among them, 12 participants were emmetropes, with a mean SER in the right eye of −0.02 ± 0.2 D (range, −0.25 to +0.25 D) and an astigmatic error of −0.12 ± 0.16 D (range, −0.25 to 0.00 D). The remaining 11 participants were myopes, with a mean SER of −1.5 ± 0.7 D (range, −0.50 to −2.50 D) and an astigmatic error of −0.38 ± 0.28 D (range, −0.75 to 0.00 D) in their right eyes.

### Corrected VA

The group mean corrected VA measured immediately prior to introduction of optical blur was −0.07 ± 0.02 logMAR. The corrected VA did not vary significantly with illumination, contrast polarity, or refractive group, and there were no significant interactions (all *P* > 0.05; Table 1).

**Table 1.** Changes in the Baseline Corrected VA and BIVAL With Contrast Polarity and Indoor Illumination

	Mean ± SEM				*P* ^*^		
	High Photopic ON	High Photopic OFF	Low Photopic ON	Low Photopic OFF	Polarity	Illumination	Polarity by Illumination
Baseline corrected VA (logMAR)
All subjects (*n* = 23)	−0.08 ± 0.02	−0.08 ± 0.02	−0.09 ± 0.02	−0.06 ± 0.02	0.320	0.706	0.304
Emmetropes (*n* = 12)	−0.12 ± 0.03	−0.11 ± 0.03	−0.12 ± 0.03	−0.07 ± 0.03	0.495^†^	0.276^†^	0.931^‡^
Myopes (*n* = 11)	−0.03 ± 0.03	−0.04 ± 0.03	−0.06 ± 0.03	−0.04 ± 0.03	—	—	—
BIVAL (logMAR)
All subjects (*n* = 23)	0.35 ± 0.03	0.40 ± 0.03	0.32 ± 0.03	0.46 ± 0.03	**<0.001**	0.345	**0.047**
Emmetropes (*n* = 12)	0.35 ± 0.04	0.39 ± 0.04	0.31 ± 0.04	0.48 ± 0.04	0.790^†^	0.968^†^	0.290^‡^
Myopes (*n* = 11)	0.34 ± 0.04	0.41 ± 0.04	0.34 ± 0.04	0.45 ± 0.04	—	—	—

* Significant *P* values are highlighted in bold.

† Two-way interaction of polarity or illumination with refractive group.

‡ Three-way interaction among polarity, illumination, and refractive groups.

### Blur-Induced VA Loss

**Figure 2.**
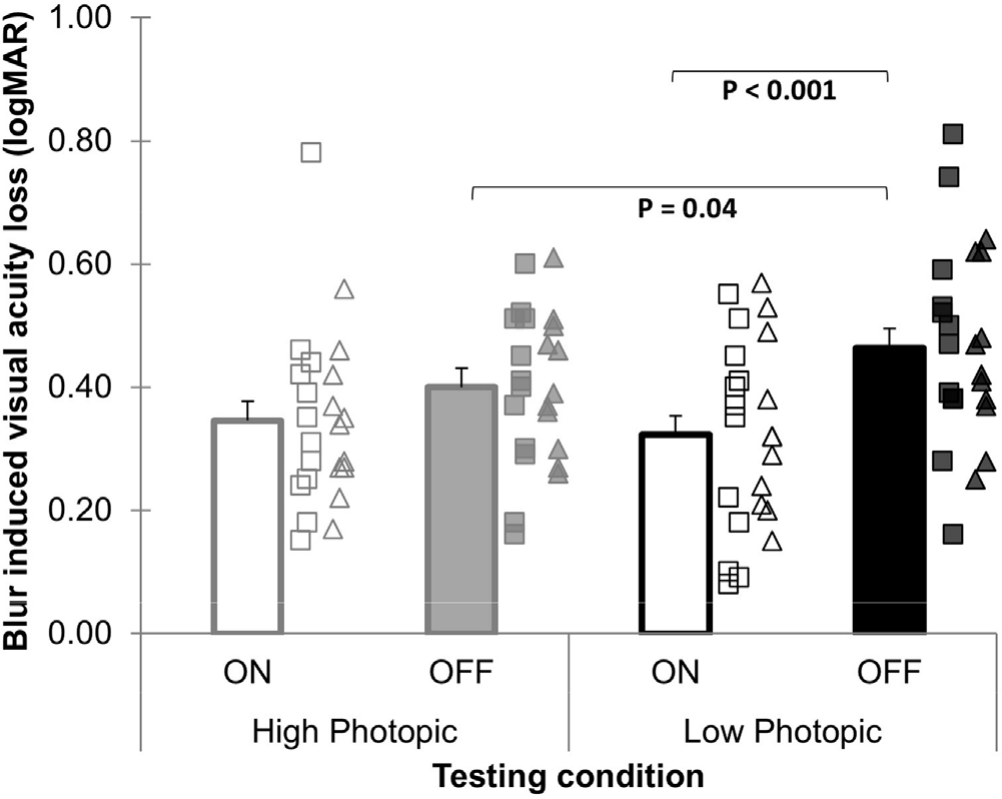
Blur-induced VA loss during ON- and OFF-pathway overstimulation in high and low photopic illumination. *Error bars* indicate the SEM. Individual changes are represented by *squares* for emmetropes and *triangles* for myopes. Greater positive values indicate greater loss in logMAR VA relative to the baseline corrected VA.

The group mean BIVAL at the start of blur was 0.38 ± 0.02 logMAR. There was a statistically significant main effect of contrast polarity, with a greater BIVAL found during OFF pathway than ON-pathway overstimulation (0.43 ± 0.03 vs. 0.33 ± 0.03 logMAR; *P* < 0.001). Although there was no significant main effect of illumination (*P* = 0.345), a significant illumination by contrast polarity interaction was found (*P* = 0.047). BIVAL was significantly greater in low compared to high photopic illumination only during OFF-pathway overstimulation (*P* = 0.04) but not during ON-pathway overstimulation (*P* = 0.45). Furthermore, the largest difference in BIVAL between the ON-pathway and OFF-pathway overstimulation was found during low photopic illumination (*P* < 0.001), whereas it was less pronounced in high photopic illumination (*P* = 0.076) (Fig. 2). These changes in BIVAL with contrast polarity and illumination were not different between emmetropes and myopes (all refractive group interactions *P* > 0.05) (Table 1).

### Relative Blur Adaptation

**Figure 3.**
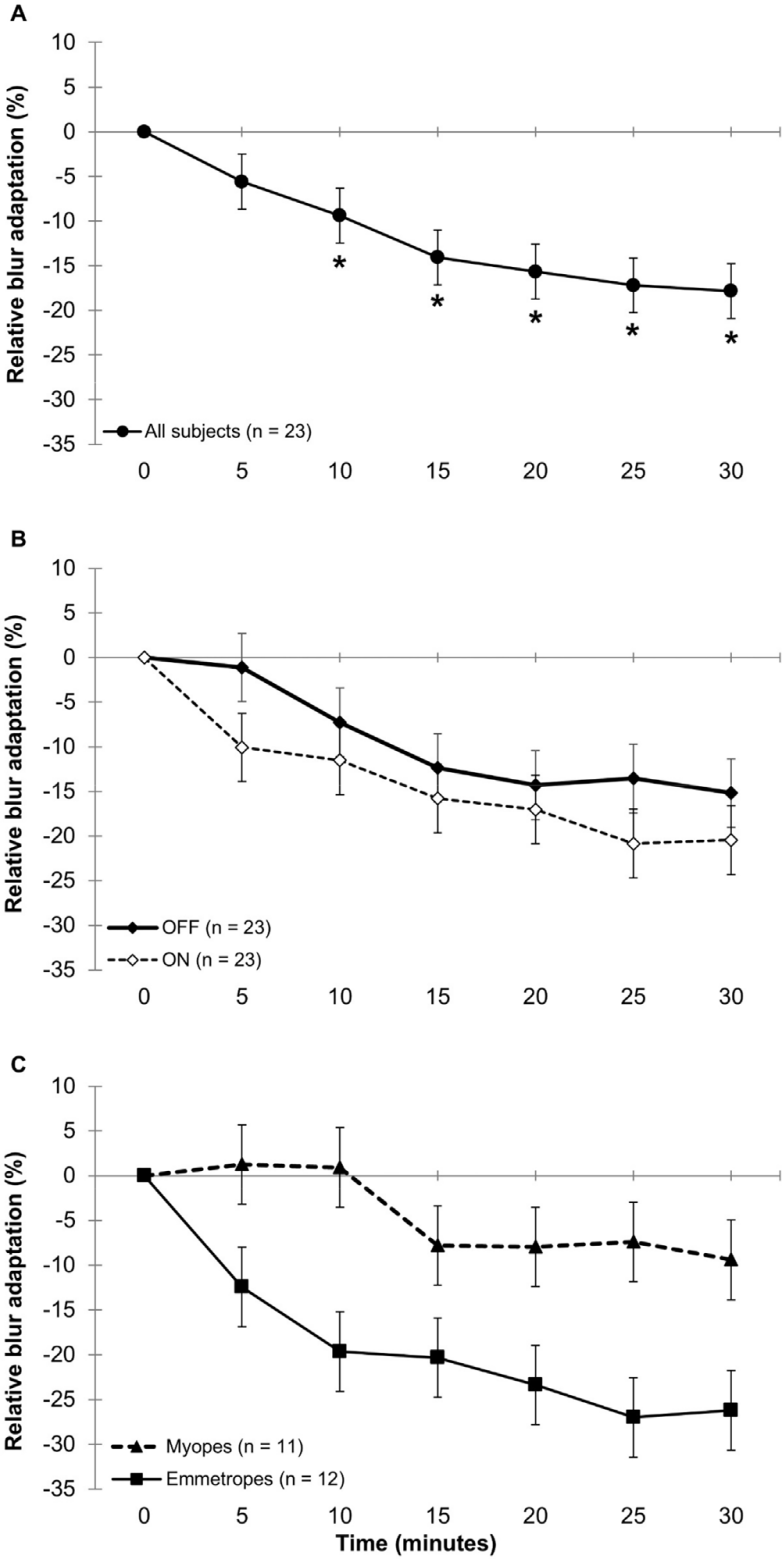
(**A**–**C**) Relative blur adaptation over time, averaged across all contrast polarity and illumination conditions in all subjects (*n* = 23) (**A**), compared between the ON-stimulating charts (*solid line*) and OFF-stimulating charts (*dashed line*) (*n* = 23) (**B**), and between emmetropes (*n* = 12, *square symbols*) and myopes (*n* = 11, *triangle symbols*) (**C**). *Error bars* indicate the SEM. Negative values indicate an improvement in VA during blur exposure relative to the initial blur-induced VA loss (BIVAL). *Statistically significant blur adaptation at the respective time point with *P* < 0.05 following Bonferroni adjustment for multiple comparisons.

A statistically significant main effect of time was found in measures of blur adaptation (*P* < 0.001), suggesting that the defocused VA, averaged across all testing conditions, improved over time relative to the BIVAL at the start of blur exposure. Compared to the baseline BIVAL, the defocused VA improved by 9% ± 3% after 10 minutes (*P* = 0.026), and 18% ± 3% after 30 minutes of blur exposure (*P* < 0.001) (Fig. 3A).

There was also a significant main effect of contrast polarity, suggesting a significantly greater blur adaptation during overstimulation with the ON pathway (−14% ± 2%) compared to the OFF pathway (−9% ± 2%; *P* = 0.01). The contrast polarity by time interaction was not significant (*P* = 0.873), suggesting a consistently greater blur adaptation during ON- compared to OFF-pathway overstimulation over the blur period (Fig. 3B). Statistically significant main effect of refractive group (*P* = 0.004) and refractive group by time interaction (*P* = 0.042) were found. In emmetropes, the first significant blur adaptation was found after 5 minutes (−12% ± 4%), whereas no significant blur adaptation was found in myopes at any time point (Fig. 3C).

**Figure 4.**
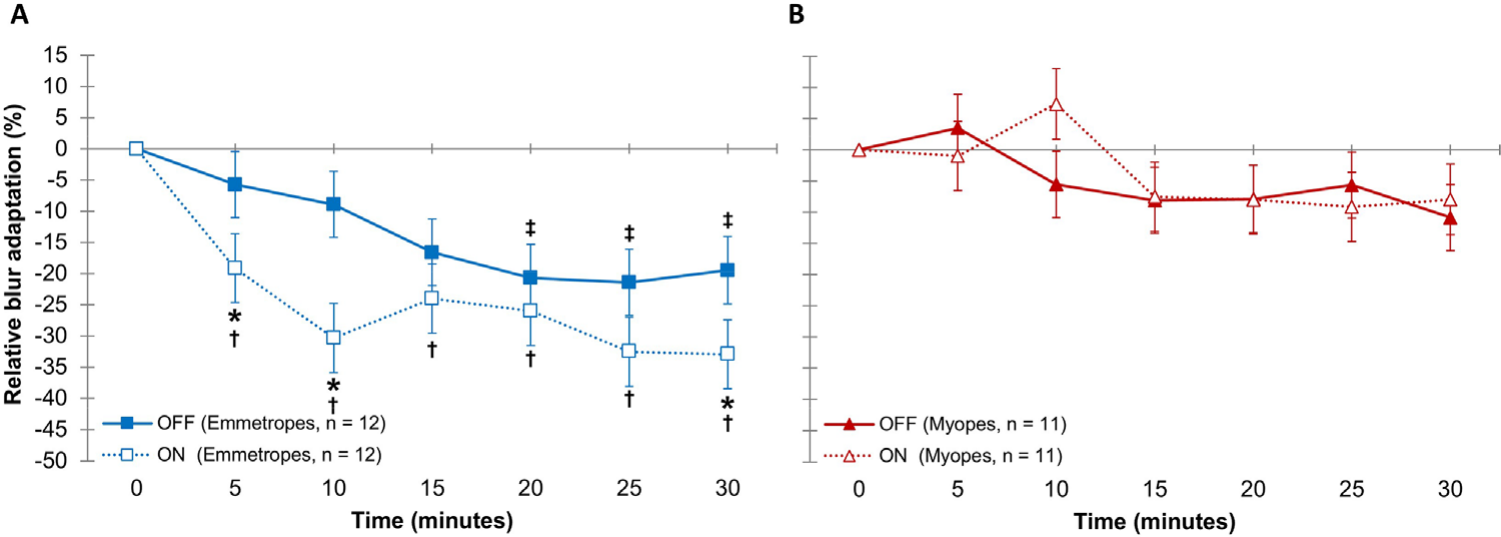
(**A**, **B**) Relative blur adaption over time during overstimulation of the ON pathway (*open symbols*) and OFF pathway (*filled symbols*) in emmetropes (*n* = 12) (**A**) and in myopes (*n* = 11) (**B**). *Error bars* indicate the SEM. Negative values indicate an improvement in VA during blur exposure relative to the initial BIVAL. Statistically significant changes, with *P* < 0.05 following Bonferroni adjustment for multiple comparisons, are indicated as follows: *difference in blur adaptation between ON- and OFF-pathway overstimulation, †blur adaptation relative to the baseline during ON-pathway overstimulation, and ‡blur adaptation relative to the baseline during OFF-pathway overstimulation.

Furthermore, a significant contrast polarity by time by refractive group interaction was found (*P* = 0.008). In emmetropes, greater blur adaptation was observed during ON- compared to OFF-pathway overstimulation after 5, 10, and 30 minutes of blur exposure (Fig. 4A). Blur adaptation was faster during ON-pathway overstimulation, exhibiting significant blur adaptation following only 5 minutes (−19% ± 5%; *P* = 0.019), whereas the first significant blur adaptation during OFF-pathway overstimulation occurred following 20 minutes (−21% ± 5%; *P* = 0.009). Myopes, however, did not show any significant difference in blur adaptation between contrast polarities or any significant blur adaptation with either contrast polarity over 30 minutes (−8% ± 6% vs. −11% ± 6%, respectively; *P* = 0.66) (Fig. 4B).

Emmetropes also showed significantly greater blur adaptation than myopes at all time points during ON-pathway overstimulation (all *P* < 0.05). However, there was no significant difference in blur adaptation between emmetropes and myopes at most blur time points during OFF-pathway overstimulation (all *P* > 0.05, except at 25 minutes with *P* = 0.041). Blur adaptation did not vary significantly with illumination or any interactions with illumination (*P* > 0.05).

### Change in Pupil Size

The baseline pupil size varied significantly with illumination and, as expected, was significantly smaller in high photopic illumination (4.8 ± 0.1 mm) compared to the low photopic illumination (6.1 ± 0.1 mm; *P* < 0.001). However, there was no significant effect of contrast polarity or refractive group or any significant interactions upon the baseline pupil size (all *P* > 0.05) (Table 2). Furthermore, the relative change in pupil size after 30 minutes of blur exposure did not vary significantly with illumination, polarity, or refractive group, with no significant interactions observed (*P* > 0.05) (Table 2).

**Table 2.** Baseline Pupil Size and Its Changes After 30 Minutes of Blur Associated With Contrast Polarity and Indoor Illumination

	Mean ± SEM				*P* ^*^		
	High Photopic ON	High Photopic OFF	Low Photopic ON	Low Photopic OFF	Polarity	Illumination	Polarity by Illumination
Baseline pupil size (mm)
All subjects (*n* = 23)	4.7 ± 0.1	4.8 ± 0.1	6.1 ± 0.1	6.0 ± 0.1	0.837	**<0.001**	0.390
Emmetropes (*n* = 12)	4.8 ± 0.2	4.8 ± 0.2	6.3 ± 0.2	6.1 ± 0.2	0.338^†^	0.688^†^	0.952^‡^
Myopes (*n* = 11)	4.7 ± 0.2	4.8 ± 0.2	6.0 ± 0.2	6.0 ± 0.2	—	—	—
Relative pupil size change after blur (%)^§^
All subjects (*n* = 23)	3.1 ± 1.7	2.3 ± 1.7	−1.2 ± 1.7	4.0 ± 1.7	0.160	0.425	0.063
Emmetropes (*n* = 12)	1.8 ± 2.3	3.1 ± 2.3	−1.2 ± 2.3	1.4 ± 2.3	0.868^†^	0.494^†^	0.150^‡^
Myopes (*n* = 11)	4.3 ± 2.4	1.5 ± 2.4	−1.2 ± 2.4	6.6 ± 2.4	—	—	—

* Significant *P* value is highlighted in bold.

^†^Two-way interaction of polarity or illumination with refractive group.

‡ Three-way interaction among polarity, illumination, and refractive groups.

§ Change relative to the baseline pupil size.

Repeated-measures ANCOVA did not reveal any statistically significant association between baseline pupil size and BIVAL (β = 0.016, *P* = 0.32), indicating that the blur-induced reduction in VA was not related to pupil size. However, a statistically significant, albeit weak, association was found between relative changes in pupil size and blur adaptation following 30 minutes of blur exposure (β = 1.03, *P* = 0.016). A 1% decrease in pupil size was associated with a 1% improvement in blur adaptation; however, only 8% of the variability in blur adaptation was explained by changes in pupil size.

## Discussion

This study revealed that VA was more resistant to blur during ON-pathway overstimulation compared to OFF-pathway overstimulation. The loss in VA associated with myopic defocus prior to blur adaptation was less pronounced during overstimulation of the ON pathway compared to the OFF pathway. In addition, more pronounced blur adaptation was found during overstimulation of the ON pathway compared to the OFF pathway. Interestingly, the observed ON pathway dominant blur adaptation was found only in emmetropes, not in myopes. Both blur adaptation and ON/OFF signaling may play a role in the mechanisms underlying myopia development. Therefore, these findings provide novel insights into the link between blur adaptation and ON-pathway overstimulation and highlight a potential deficiency in ON-pathway–mediated blur adaption in myopic eyes.

### Role of ON/OFF Overstimulation in Resolution With Blur

The current study provides evidence regarding the contribution of ON/OFF pathways to psychophysical measurements of VA under optical blur conditions, while controlling for other confounding factors. The mean corrected VA, averaged across all experimental conditions, was −0.07 logMAR and was reduced by 0.38 logMAR (approximately four lines) immediately following the introduction of 1 D myopic defocus. The loss in VA due to blur varied with preferential stimulation of the ON/OFF pathways. Exposure to myopic defocus during ON-pathway overstimulation resulted in one-line less worsening in VA compared to OFF-pathway overstimulation, suggesting an interaction between ON/OFF-signaling pathways and resolution in the presence of blur in central vision.

Previous studies comparing uncorrected VA between standard and reversed contrast polarity charts have yielded various findings, including better VA with standard polarity charts (OFF-pathway overstimulation),^11^^,^^15^^,^^16^ no difference in VA between the two chart polarities,^12^^–^^14^ and a better VA with reversed polarity charts (ON-pathway overstimulation).^7^^–^^11^ Reports of improved uncorrected VA found during OFF-pathway overstimulation could be explained by the smaller pupil size and the increased depth of focus associated with greater overall luminance of a chart displaying black letters on a white background compared to the reversed polarity chart with white letters on a black background.^11^^,^^15^^,^^16^ However, when the confounding effect of chart luminance is minimized, as in the current study, better VA was found with reversed polarity charts. This may be explained by optical factors related to the substantial increase in the Weber contrast associated with the prevailing dark background surrounding the light targets in the chart compared to the prevailing light background surrounding the dark targets in the standard polarity charts.^9^^–^^11^ The advantage in Weber contrast during ON-pathway overstimulation compared to OFF-pathway overstimulation becomes even more pronounced when retinal image quality is reduced, leading to improved VA during ON-pathway overstimulation with reversed polarity charts in conditions such as age-related increase in ocular light scatter,^10^ uncorrected refractive errors,^7^^,^^8^ or induced optical defocus, as in the current study. Interestingly, when the influences of image blur, ocular aberrations, and overall chart luminance are minimized, VA measured during OFF-pathway overstimulation tends to outperform VA measured during ON-pathway overstimulation, particularly at higher matched luminance levels.^11^ The improved VA during OFF-pathway overstimulation than ON-pathway overstimulation observed under aberration-free conditions is thought to be primarily caused by an increased response saturation with greater neuronal blur involved in ON-pathway signaling.^16^^,^^56^ The current study did not provide aberration-free viewing conditions to replicate these findings; rather, it measured VA under induced myopic defocus.

The greater blur-induced loss in VA during OFF- than ON-pathway overstimulation found in the current study is also consistent with a previous study reporting on increased subjective perception of blur when digitally blurred images were viewed in which the high spatial frequencies were attenuated to a greater extent in the OFF channel than in the ON channel.^57^ In the human retina, the OFF ganglion cells outnumber the ON cells and branch more densely with smaller receptive field sizes, thus rendering the OFF pathway more susceptible to image blur.^3^^,^^58^

Collectively, these results indicate that the ON-pathway signaling exhibits greater resistance to image blur, suggesting that the degradation of small details in the visual scene via optical defocus or light scatter would result in a greater deterioration of central vision for OFF stimuli compared to ON stimuli. Therefore, the myopiagenic effects of image blur, known as form-deprivation myopia, may be suppressed through ON-pathway signaling but enhanced through OFF-pathway signaling, an effect that has been reported in animal studies.^40^

### Blur Adaptation

A significant improvement in defocused VA was found during the 30-minute period of exposure to 1 D myopic defocus, consistent with blur adaptation. Across all viewing conditions, the first significant improvement occurred after 10 minutes, with VA improving by 9% (corresponding to ∼0.04 logMAR change). This improvement continued progressively, reaching an 18% increase in VA (corresponding to ∼0.08 logMAR change) after 30 minutes of blur exposure. The observed magnitude and time course of overall blur adaptation are consistent with previous research, which has reported a 0.06 to 0.16 logMAR improvement in VA following a similar 30-minute exposure period to 1 D myopic defocus.^18^^,^^22^^,^^23^^,^^59^ Additionally, an earlier study found a significant improvement in defocused VA within the first 4 minutes of blur exposure.^23^ These studies therefore suggest that, after only a brief period of blur exposure, partial compensation of the initial blur-induced decline in VA occurs via blur adaptation to restore vision. However, whether blur adaptation is influenced by the differential stimulation of ON/OFF pathways remains largely unresolved in these past studies.

### Role of ON/OFF Overstimulation in Blur Adaptation

Spatial frequency-selective contrast adaptation occurring in the retina and brain plays a key role in the mechanisms underlying blur adaptation.^18^^,^^26^ The current study offers further insights into these mechanisms. The improvement in VA with blur adaptation was greater during ON-pathway overstimulation, showing a 14% improvement in defocused VA after 30 minutes of blur exposure, compared to a 9% improvement in defocused VA observed during OFF-pathway overstimulation. Sato et al.^60^ demonstrated that suprathreshold contrast adaptation was also selective to contrast polarity. They found that contrast sensitivity decreased for ON- and OFF-stimulating texture patterns following exposure to patterns with the same polarity, but not those with reversed polarity.

Additionally, they found polarity asymmetry in suprathreshold contrast adaptation, with OFF stimuli inducing greater contrast adaptation than ON stimuli. These findings indicate that polarity-selective mechanisms are involved in adaptive responses to both focused stimuli with suprathreshold contrast and defocused stimuli with reduced contrast. Although adapting to OFF-polarity contrast has a greater effect on the subsequent perception of suprathreshold contrast under no blur conditions, the current findings suggest that adapting to ON stimuli has a more significant impact on the subsequent perception of blurred images with near threshold contrast, particularly at higher spatial frequencies.

The ON/OFF asymmetry in the magnitude of blur adaptation observed in this study is a novel finding, although the exact underlying mechanisms remain unclear. Because blur adaptation is primarily driven by adaptation to contrast^17^^,^^28^^,^^61^ and evidence indicates greater contrast sensitivity in the ON pathway compared to the OFF pathway,^44^^,^^62^^–^^64^ it is reasonable to speculate that the blur-induced decline in image contrast is processed more effectively during ON-pathway overstimulation, leading to a more pronounced adaptation response. Stronger ON-pathway responses have also been found under reduced lighting at a given contrast level,^44^ which may explain the relatively greater ON-dominant blur adaptation observed under low photopic lighting in the current study.

There is also compelling evidence that retinal dopamine, a key neuromodulator influencing visual function,^35^^,^^47^^–^^49^ is released primarily by the dopaminergic amacrine cells that receive excitatory input from cone-mediated ON bipolar cells.^47^^,^^50^^,^^65^^–^^67^ Retinal dopamine alters the coupling of the horizontal and amacrine cells and changes receptive field sizes, potentially contributing to changes in VA.^50^^,^^68^ Additionally, short-term exposure to computer-generated ON stimuli increases retinal dopamine in animal models.^41^ Although retinal dopamine may also be elevated by exposure to myopic defocus,^69^ most other research indicates that it could be downregulated by both myopic and hyperopic defocus,^70^^,^^71^ potentially in response to changes in the contrast and spatial frequency content of the image.^72^ Therefore, the current findings suggest that the ON/OFF pathways play a significant role in blur adaptation, with overstimulation of the ON pathway leading to a greater improvement in VA during blur adaptation, possibly due to elevated retinal dopamine levels. Considering that rod photoreceptors are also involved in upregulation of dopaminergic amacrine cells,^73^ the current finding of ON/OFF pathway contributions to blur adaptation may also provide a possible explanation for the observed blur adaptation in the rod-dominated parafovea, independent of foveal vision.^25^^,^^59^

### Myopia Is Associated With Deficient ON-Dominant Blur Adaptation

In this study, the emmetropes exhibited greater blur adaptation than myopes during ON-pathway overstimulation. Moreover, only emmetropes showed a more pronounced blur adaptation with ON- compared to OFF-pathway overstimulation. These results suggest that ON-pathway dominance in blur adaptation is a normal response in emmetropic eyes, whereas this ON-dominant blur adaptation response appears to be deficient in myopic eyes. Growing evidence indicates that myopia is associated with ON pathway deficiencies in both humans^43^^–^^45^ and animals.^39^^,^^40^ Retinal electrophysiological recordings in humans show that emmetropes have higher sensitivity to high-contrast ON stimuli compared to OFF stimuli,^45^ whereas myopes exhibit weaker responses to high-contrast ON stimuli^45^ and lower sensitivity to low-contrast ON stimuli compared to OFF stimuli.^44^ Further research is required to determine whether the observed ON-pathway deficiency in the blur adaptation response among myopes precedes or results from myopic eye growth.

Previous studies on blur adaptation have measured VA using charts with standard contrast polarity, primarily stimulating the OFF pathway, and reported either greater blur adaptation in myopes than emmetropes^20^^,^^22^ or no significant difference between the two groups.^21^^–^^23^ Similarly, the current study found no significant difference in blur adaptation between refractive groups at the majority of time points during OFF-pathway overstimulation. It is important to note that the study included only myopic participants who were optimally corrected with single-vision spectacle or contact lenses worn full time, thereby reducing the likelihood of habitual blur exposure and limiting further blur adaptation. Additionally, both emmetropes and myopes showed a similar magnitude of blur-induced VA loss before adaptation, suggesting that any prior blur adaptation had little influence on the VA measurements taken before and during adaptation to blur in this study.

All myopic subjects were corrected only by single-vision spectacles on the examination days. This may have affected the performance during VA assessments for those myopic subjects who were habitually corrected with soft contact lenses. However, any impacts on visual performance are expected to be minimal, particularly given that the study did not find any significant difference in VA between emmetropes and myopes prior to and at the start of exposure to blur and during blur adaptation while the OFF pathway was overstimulated. Furthermore, this study did not measure the corrected VA following blur adaptation which could have provided additional insights into the aftereffects of blur adaptation associated with overstimulation of the ON/OFF pathways, highlighting the need for future studies. In addition, because either the OFF or ON pathway is overstimulated during the assessment of VA using standard or reversed polarity charts, a limitation of this study is the lack of inclusion of a control condition to isolate the sole effect of inducing blur without additional ON/OFF-pathway overstimulation upon VA and blur adaptation. Future studies could address this limitation by measuring the VA using methods that provide balanced stimulation of both ON and OFF pathways, such as by employing square-wave gratings with balanced contrast polarity or custom-designed VA charts featuring optotypes constructed from random noise patterns or presented with temporally alternating contrast polarity relative to the background.

### Role of Pupil Size in Blur Adaptation

The group mean pupil size did not change significantly from baseline during blur adaptation. Although the individual reductions in pupil size over 30 minutes were weakly associated with greater adaptation to blur, they accounted for only 8% of the variability observed in the blur adaptation. Therefore, the overall improvement in VA following blur exposure was primarily caused by neural mechanisms, with only minimal contribution from optical factors related to increased depth of focus and decreased ocular aberrations from reduction in pupil size.^17^^,^^19^^,^^22^

## Conclusions

The ON/OFF pathways were found to play a significant role in resolution with blur and blur adaptation. VA deteriorated less from myopic optical blur during ON-pathway overstimulation compared to OFF-pathway overstimulation, with no difference between emmetropes and myopes. The blur adaptation response, however, was enhanced during ON-pathway overstimulation compared to OFF pathway overstimulation, an effect that was only observed in emmetropes, not in myopes. These results suggest a deficient blur adaptation in the ON pathway in myopic eyes, and further research is warranted to investigate whether it is a cause or consequence of myopia.
